# A pilot community-based Diabetes Prevention and Management Program for adults with diabetes and prediabetes

**DOI:** 10.1017/cts.2024.623

**Published:** 2024-10-29

**Authors:** Ranjita Misra, Samantha Shawley-Brzoska

**Affiliations:** 1 School of Public Health Professor, Department of Social & Behavioral Sciences, Robert C Byrd Health Science Center West Virginia University, Morgantown, WV, USA; 2 School of Public Health Research Assistant Professor, Department of Social & Behavioral Sciences, Robert C Byrd Health Science Center West Virginia University, Morgantown, WV, USA

**Keywords:** Behavioral intervention, diabetes mellitus, prediabetes, prevention, self-management, rural

## Abstract

**Background:**

West Virginia is a rural state with high rates of type 2 diabetes (T2DM) and prediabetes. The Diabetes Prevention and Management (DPM) program was a health coach (HC)-led, 12-month community-based lifestyle intervention.

**Objective:**

The study examined the impact of the DPM program on changes in glycosylated hemoglobin (A1C) and weight over twelve months among rural adults with diabetes and prediabetes. Program feasibility and acceptability were also explored.

**Methods:**

An explanatory sequential quantitative and qualitative one-group study design was used to gain insight into the pre- and 12-month changes to health behavior and clinical outcomes. Trained HCs delivered the educational sessions and provided weekly health coaching feedback. Assessments included demographics, clinical, anthropometric, and qualitative focus groups. Participants included 94 obese adults with diabetes (63%) and prediabetes (37%). Twenty-two participated in three focus groups.

**Results:**

Average attendance was 13.7 ± 6.1 out of 22 sessions. Mean weight loss was 4.4 ± 11.5 lbs at twelve months and clinical improvement in A1C (0.4%) was noted among T2DM adults. Program retention (82%) was higher among older participants and those with poor glycemic control. While all participants connected to a trained HC, only 72% had regular weekly health coaching. Participants reported overall acceptability and satisfaction with the program and limited barriers to program engagement.

**Conclusion:**

Our findings suggest that it is feasible to implement an HC-led DPM program in rural communities and improve A1C in T2DM adults. Trained HCs have the potential to be integrated with healthcare teams in rural regions of the United States.

## Introduction

The high health and economic burden of type 2 diabetes (T2DM, 16.2%; ranked 1^st^ in the nation) and pre-diabetes (34.8%) in the predominantly rural Appalachian state of West Virginia (WV) [1] necessitates access to prevention and diabetes self-management education and support (DSME/S). The higher prevalence of diabetes parallels other chronic conditions (e.g., obesity, hypertension, and cardiovascular disease) that increase health risks and complications [2]. Contributing factors include suboptimal social determinants of health [3], geographical isolation [4], and lack of access to healthcare and (DSME/S) programs that are vital to maintaining a healthy lifestyle and optimal glucose management [3,5]. The majority (91%) of West Virginians live in rural, medically underserved counties (91%) where health services and interventions to address diabetes disparities are limited [6].

Evidence-based interventions in community settings such as churches and YMCAs, are recognized for providing programs to address health disparities. Weight loss and lifestyle modifications (e.g., healthy diet and regular physical activity) among overweight and obese T2DM and pre-diabetes adults reduce glucose levels and risk for cardiovascular disease [7,8]. Yet, one in two patients with diabetes have poor glycemic control [9,10]. Adherence to healthy lifestyle and diabetes self-care regimen, important for long-term metabolic control and improved quality of life, are critically lacking [11] with a high economic burden of diabetes in WV due to disability, time lost from work, and premature death [12]. Poor disease coping, mental well-being, and depression impede self-care regimen and adherence behaviors [13,14].

Traditionally, individuals with diabetes and prediabetes have different educational protocols. However, the current Diabetes Prevention and Management Program (DPM) combined two evidence-based programs – The National Diabetes Prevention Program (NDPP)[15] and the Association of Diabetes Care and Education Specialists (ADCES7) self-care behaviors [16]. The NDPP provides a prime example of interventions for diabetes risk reduction. In addition, other diabetes self-management programs (e.g., Look AHEAD study) also showed that obese T2DM adults benefit from weight loss and lifestyle modifications [17]. Both programs have been successfully translated to community settings [18]. Since both NDPP and ADCES use non-pharmacological lifestyle intervention, there is compelling reason to combine them for diabetes risk reduction and management in resource-poor settings. However, to our knowledge, no program has targeted both T2DM and prediabetes adults, offered free to participants, and in a community setting in rural WV.

The Diabetes Prevention and Management (DPM) program was a 12-month multicomponent behavioral intervention that focused on knowledge, skills, behavior modification strategies, and weight loss in rural adults with prediabetes and T2DM. It was first implemented, using a culturally tailored NDPP and ADCES7 curriculum in two large community trials in rural India [19,20]. For this study, we adapted the DPM program content and delivery, in partnership with stakeholders, using a collaborative process that allowed the innovative adaptation of program components to address the perceptions of disease, health behavior, and how to use available community assets and resources to promote behavior change. We used the community-based participatory research (CBPR) approach for Appalachian culture and available resources [21]. Diverse stakeholders included a WV church advisory board (CAB), patient partners, family members, service providers, and experts in nutrition, public health, and behavioral medicine. The program design and implementation were informed by health behavior change theoretical frameworks [22].

The study examined the impact of the DPM program on changes in glycosylated hemoglobin (A1C) and weight loss over twelve months among rural adults with diabetes and prediabetes. Program feasibility (based on recruitment, retention, and intervention metrics) and acceptability (based on program engagement, satisfaction, and completion rate) of implementing the DPM intervention in community settings (churches) were also explored.

## Methods

### Study design and participants

The study used an explanatory sequential quantitative and qualitative one-group design to gain insight into the pre-and 12-month changes in participant’s health behavior and clinical outcomes. Qualitative focus groups provided additional feedback on program experience, satisfaction barriers/enablers for behavior change, and program engagement. While there are limitations to using a one-group design, the 12-month intervention did not allow for a wait-listed or usual care group that is commonly used as a comparator to examine intervention effectiveness. Hence, baseline assessments served as the comparator for changes in 6- and 12-month program outcomes. Data was collected in 2016–2018 from two cohorts of participants (*n* = 94) who joined the program sequentially in 2015–17[23]. Recruitment procedures included using flyers/announcements, informational meetings, and newspaper advertisements. Eligibility included age 18 years and older, overweight or obese status (body mass index [BMI] ≥ 25 kg/m^2^), and a diagnosis of prediabetes or T2DM. The study was conducted according to ethical guidelines and all procedures for this research study were approved by the Institutional Review Board at a large public university. All participants provided written informed consent prior to their baseline assessment and program participation. In addition, all participants were invited to participate in focus groups; twenty-two individuals volunteered. Three focus groups were conducted by two trained qualitative researchers who consulted on the project. Participants provided qualitative feedback about the overall program and experience.

### The DPM intervention

The DPM program’s 22 sessions included 16 core sessions (6 months) and 6 post-core sessions (6 months). The curriculum was adapted from the NDPP[15] and ADCES7 for health behavior modifications to prevent and manage T2DM [16]. The intervention was adapted and culturally tailored for West Virginia rural communities in all aspects including information gathering, preliminary design, testing, refinement, and final implementation to address the unique characteristics of the Appalachian population, utilizing the knowledge of experts, lived experience of patients and vetted by the CAB. In addition, the CBPR principles[24] and ADAPT-ITT model[25] guided the adaption and tailoring of program components 10 months prior to the program launch. As indicated earlier, engaging diverse stakeholders collaboratively allowed DPM program components to address the perceptions of disease and self-care in Appalachia, empower individuals for behavior modifications, improve awareness of psychosocial factors and barriers to behavior modifications, health coaching and support from peers on how to address complex disease self-care, and how use of available community assets and resources to promote behavior change goals.

Since WV is the 3^rd^ most rural state in the country and 90% of the counties are medically underserved, individuals have limited access to healthcare providers, fresh food, and local gyms/nature trails. The DPM curriculum incorporated the Appalachian dietary practices to maximize nutritional value with minimal financial burden. In addition, dietary sessions incorporated local vegetables specific to the region (e.g., cushaw squash, pawpaw, sweet potatoes, and green beans), cut fat/calories, and improved nutritive value through cooking demonstration, potlucks, etc. Many historical studies have suggested the Appalachian culture has both negative (e.g., fatalism) and protective (e.g., strong social family ties, religion, and faith) factors that impact health behavior and outcomes [13,26–28]. Hence, participants were encouraged to use social support for reinforcement of health behavior changes and sustainability. The stakeholders provided valuable feedback to the researchers that promoted co-learning and empowering processes that were sensitive to the needs of the target population. In addition, it allowed for refining the program content for cultural context, study measures, and data collection procedures that were sensitive to the needs of the target population. The mutual ownership of the process and program implementation was crucial for the delivery of this culturally acceptable pilot DPM program [29].

The cultural adaptation and tailoring of the intervention also incorporated the key principles of CBPR[24] such as local assets and resources, ecological perspectives, co-learning and balance between research and practice, and equitable partnerships that shaped the study design and implementation strategies. While these principles served to increase the engagement of stakeholders to reduce diabetes disparities, we utilized this process and community network in other rural settings for a meaningful engagement for program planning, implementation, and evaluation [19,20]. The process and input resulted in the finalized study design, recruitment strategies, data collection and dissemination, and program curriculum built on mutual learning, trust building, and evidence-informed solutions.

The intervention was modeled after the NDPP and included 60-minute group educational sessions for 12 consecutive weeks, biweekly sessions for two months, and monthly sessions for the last six months. Trained health coaches (HCs) delivered the educational sessions and provided weekly health coaching to participants. Each participant was assigned an HC who assisted with goal setting and weekly follow-up to identify behavior modification goals and review strategies (average of 5–10 minutes) via phone calls or use emails and texts (based on participant preference). These discussions provided the opportunity to answer questions, provide continuous feedback on initiation and maintenance of health behaviors, identify easy and pragmatic ways to make healthier short-term goals, and reinforcement of health education messages. Two cooking demonstrations were part of the educational sessions, were interactive, and included taste-testing and skill-building exercises that emphasized key concepts from the dietary educational sessions. It also provided helpful tips, substitutions for ingredients, recipes, food safety, and nutritional information. The church offered a large kitchen, and an open hall for delivery of the educational program; one also had a gym for physical activity sessions. Weekly sessions included weigh-ins, group sharing, goal setting, and problem-solving skills for healthy behavior changes.

The program also encouraged participants to keep daily food and activity logs. Additionally, HCs provided written (tailored) feedback for those who submitted food and activity logs recognized positive changes, and provided general encouragement. Participants received self-help educational materials, a CalorieKing Calorie, Fat & Carbohydrate Counter book [30], and a pedometer. The program was implemented in two churches in two geographically separated large counties to ensure easy access to participants. In addition, weekday evening hours (5:30 to 6:30 pm) or weekend afternoon (1:30 to 2:30 pm) allowed the majority of participants to attend the educational sessions. All sessions were video-recorded, and participants who missed the session were provided a closed YouTube link.

### Theoretical framework

Two health behavior theories were used to guide the tailoring of the DPM program for the Appalachian culture and rural residents. It was a critical part of the planning process. The Social Cognitive Theory [22] was used as the primary behavioral change theory with Self-Determination Theory [31] providing additional cultural context for health behavior change and outcomes. Both theories demonstrate the phases of behavior modification, the social environment in which an individual performs the behavior, and experiences that shape the decision-making abilities for diabetes prevention and self-care for glucose control and favorable health outcomes [32,33]. Similar to prior evidence-based interventions, the program focused on enhancing participant’s knowledge about diabetes, behavior change intentions and goal setting, action planning, self-monitoring, and discussion with HCs to take care of their health and make informed lifestyle decisions. Self-efficacy, a central tenet of both theories, is the confidence in the ability to successfully perform a behavior for behavior initiation and maintenance [32,34]. A combination of improved self-efficacy and self-care purported to assist participants with behavior change techniques that were integrated for health behavior changes and outcomes [35,36]. Participants’ barriers and enablers for behavior change and program engagement were also assessed post-program, qualitatively, to guide further fitting of the intervention into WV rural communities.

### DPM implementation strategies

The implementation strategies were chosen based on literature review [37] and by utilizing the knowledge of experts (interventionists, service providers, diabetes researchers, and healthcare providers), patients, and the CAB. These strategies included adapting and tailoring educational materials to include locally available resources such as locally available and low-cost foods and free/low-cost physical activity resources (e.g., discounted gym or YMCA membership through health insurance, walking in the high school field, church, or trails). In addition, educational materials and lessons were tailored for participants with low health literacy. Food demonstrations reinforced locally available and seasonal foods. Potlucks allowed participants to share their recipes. Weekly health coaching was included to improve interpersonal interactions and relationships as well as address barriers to health behavior change and program engagement. These strategies were chosen to enhance adoption, implementation, and sustainability of health promotion behavior. All strategies were vetted by the CAB to guide fitting them for rural individuals, reduce participant burden, and allow assessments to track program feasibility, satisfaction, and changes in program outcomes, both quantitatively and qualitatively.

Similar to prior interventions that have used intervention acceptability and feasibility measures [25,38], we defined feasibility as the extent to which the DPM intervention can be carried out in the rural Appalachian setting & acceptability as the perception that the program was acceptable by individuals with diabetes and prediabetes. Hence, the feasibility metrics measured for effectiveness were the number of participants successfully enrolled (recruitment), proportion of participants who were assigned and successfully connected with HCs for their weekly health coaching, as well as duration of their interaction every week (at least 5 minutes).

### Health coach training and program fidelity

The training of HCs has been described in detail elsewhere [21]. Briefly, HCs completed a 20-hour training provided by a multidisciplinary team using the culturally adapted DPM curriculum. HCs included were students enrolled in professional programs such as Public Health, Nursing, Pharmacy, Medicine, Physical Activity and Sports Sciences, Exercise Physiology, and Human Nutrition. The training familiarized them with the curriculum, delivering the educational sessions, health coaching, and data collection measures. Program sessions focused on healthy lifestyle changes, knowledge, session content, research ethics, review of diet and activity tracking logs, and how to address participant challenges and needs. The intervention fidelity and program outcomes remained aligned with the two original programs. Intervention fidelity included audits to examine the communication pathways between the HCs and the participants. Fidelity audits, using direct observation and a standardized checklist, ensured HCs adhered to the same protocol for each educational session. In addition, HCs completed a mock educational session with their peers and received constructive feedback for improvements prior to their educational sessions. There was a 96% adherence to the standardized fidelity checklist for the current program. The program leader and study coordinator were responsible for guiding HC challenges to participant interactions during weekly discussions to maximize intervention fidelity. In addition, the study coordinator completed random evaluation of HC calls for quality and fidelity.

### Data procedures and data collection

Baseline, 6-month, and 12-month assessments (clinical, anthropometric, and behavioral measures) were collected at the intervention sites from 7 am to 10 am. The participants were instructed to fast for 8–10 hours and trained phlebotomists collected fasting serum blood for labs. HCs completed blood pressure and anthropometrics assessments that included weight, height, and waist circumference in a private setting. Clinical measures included A1C, weight, and blood pressure. Surveys were completed by participants, but HCs assisted with surveys as needed. Clinical laboratory testing was provided by the Mon General Hospital consistent with established standards. A $25 gift card to a local grocery store was given as incentive for program evaluation/surveys with each assessment completion.

## Measures

### Feasibility

The primary outcome for this pilot trial was feasibility, which was determined by the following: number successfully enrolled (recruitment); proportion of participants who were assigned and successfully connected with HCs for their weekly health coaching (via phone calls, emails or texts), as well as adequate HC discussion time > = 5 minutes) were included in the feasibility metrics. Mean number of days to connect participants to an HC; and number of DPM program sessions completed. The proportion of participants with an A1C test at 6- and 12-month program assessment was used to measure retention. All participants were assigned a HC for their weekly health coaching at the first day of the program. Feasibility metric and how each was calculated is provided in Table 1. Proportion of participants who successfully connected to a trained HC within 2 weeks was included as feasibility metrics for program engagement.

**Table 1.** DPM feasibility metrics and results

Goal	Definition/Calculation	Intervention Program
Recruit 100 eligible participants over 6 months	Number of eligible participants enrolled/ total number of eligible patients	Recruited 94 participants over 6 months
Successfully connect 75% or more participants to a HC	# of participants with at least one encounter (via phone or text) with a HC /total number of participants enrolled in the program	100% participants connected to a HC
Connect participants to HC in 7–14 days, Mean (SD)	Mean # of days between date of assignment to date of first encounter with a HC across study sites	5.5 (4.6) days to connect to a HC
At least 70% of participants connect with HC for weekly discussion (> = 10 minutes)	# of participants with at least one encounter (via phone or text) with a HC for at least 10 minutes or detailed text with queries and response/ total number of participants enrolled in the program	72% participants connected to a HC for weekly discussion
Participants complete at least 50% of DPM sessions	Number of DPM sessions the Health Coach successfully delivers to program participants	63 participants completed at least 50% of the sessions (67%); 37 (33%] completed 17 or more (75%) of all 22 sessions
At least 80% of participants with 6-month A1C	# of participants with an A1C test at 6-month follow-up/ # of enrolled participants	83.3% A1C test at 6 months
At least 80% of participants with 12-month A1C at the end of study	# of participants with an A1C test at 12-month follow-up/ # of enrolled participants	82.2% A1C test at 12-months

HC = Health Coach; DPM = Diabetes Prevention and Management Program; A1C = Glycosylated hemoglobin level.

### Acceptability

A benefits, barriers, and satisfaction survey [39] examined participants’ perception of program benefits and satisfaction at the end of the program. Focus groups at 12-month assessed program acceptability, experience of working with trained HCs, and program completion.

### Program attendance and tracking

Program attendance was measured by calculating the mean number of sessions attended (range 1–22 sessions) to determine the dose-response relationship between program attendance and program outcomes. In addition, participants were dichotomized as consistent attendance (> = 75% of program sessions) versus if they attended at least 50% of educational sessions. Program completers were dichotomized as attending sessions after the first month and completed all assessments, or non-completers with < = 4 educational sessions and completed only baseline assessment, respectively.

### A1C

Glycosylated hemoglobin or A1C was assayed at Mon General Hospital at baseline, 6- and 12-months; the lab met the national standards for high-quality testing procedures.

### Weight

Weight was measured using a calibrated electronic Seca (Chino, California) digital scale. Height was measured with the participant’s head positioned in the horizontal plane. BMI was calculated from height and weight measured at each assessment. Participants were weighed at baseline and at each session by an HC. Average weight change between baseline, 6- and 12- months was calculated.

### Program benefits and barriers survey

Program benefits, satisfaction, and barriers were assessed with a 33-item survey questionnaire developed by the researchers [39]. The survey (Likert scale) examined participant’s benefits of program participation (7 items), barriers to session attendance (5 items), and overall program satisfaction (1 item), post-program. The survey items were summed for benefits, barriers (reverse coded), and satisfaction scores with higher scores indicating higher benefits and overall satisfaction, and lower barriers. In addition, open-ended questions provided the opportunity to respond to specific benefits and barriers and identify the most interesting components that lead to health behavior improvements.

### Qualitative focus groups

Three focus groups were conducted with a subsample of twenty-two participants from both sites/cohorts by two trained qualitative researchers who consulted on the project. A greater number of participants was aimed to reach saturation and to understand their experiences, satisfaction, and benefits of participation, and barriers to attendance, lifestyle changes, and program completion. A protocol was used to guide discussion, which prompted participants to share their feedback about the program and ask about how participating in DPM impacted their dietary habits and behaviors. Focus groups lasted 90 minutes and were audiotaped and transcribed verbatim.

### Sample size

**Figure 1.**
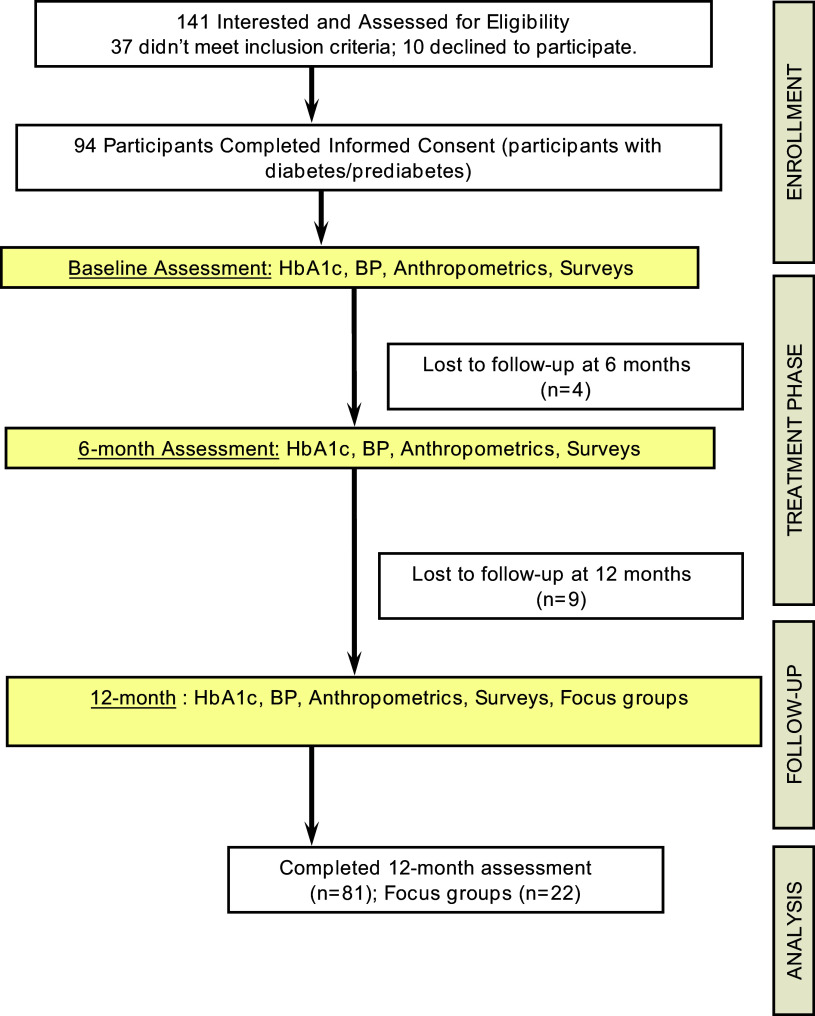
CONSORT diagram.

Our a priori sample size calculation indicated an enrollment of 90 participants. We accounted for a 20% dropout rate to provide a clinically meaningful change in our primary outcome (A1C, 0.4 at 12 months) with 80% power. A convenience sample of eligible adults was screened and enrolled in the program from 2015–2017 (see Figure 1). However, nine participants dropped out in the first three weeks due to work/family/travel-related conflicts.

### Statistical and qualitative analysis

All quantitative analyses were performed using SPSS software, Version 29 for Windows. Program outcomes (A1C and weight) and program acceptance (benefits, satisfaction, and barriers) were reported as mean ± SD. Percentages were calculated for the categorical variables. An intention-to-treat analysis was used for program outcomes. Mean change in weight was assessed over 12 months separately by gender, T2DM vs prediabetes status, and program attendance. We examined the proportion of participants with an A1C < 8% at follow-up as well as mean change in A1C in each group with an A1C test at follow-up. Association between program outcomes, number of sessions attended, and sociodemographic characteristics were assessed using Pearson’s correlation. Statistical inferences were based on a significance level of *p* ≤ 0.05.

*Focus group data:* The focus group audio recordings were transcribed verbatim and coded in NVivo by two trained research assistants to ensure content accuracy. The validity of the study was established by using two trained coders and triangulation of codes agreed upon by the team to improve reliability and intercoder agreement (e.g., kappa coefficients). Content accuracy and thematic analysis were related to program participation benefits, satisfaction, and barriers. The coders used a hybrid inductive and deductive (or “theoretical”) coding approach to categorize major thematic categories and sub-categories related to intervention acceptability and satisfaction.

## Results

Figure 1 presents the Consolidated Standards of Reporting Trails (CONSORT) Diagram. Out of 141 potentially interested participants, 107 were screened as eligible and 94 individuals provided consent for enrollment and completed the baseline assessment for participating in the DPM program. Table 2 presents the baseline characteristics of the 94 participants who enrolled in the program. Mean age was 58.8 ± 12.3 years (range 20–83 years) and 73% were females. The sample was 93.1% non-Hispanic Whites, reflective of the racial and ethnic makeup in the state (97% NHWs). The majority (60%) of the participants had T2DM, had a family history of diabetes (80.6%), were employed full-time (69.3%), didn’t have a college or professional degree (30%), and reported a family income of less than $50,000 (61.1%). The number of individuals who lived in the household ranged from 0 – 5 with an average of 2.1 ± 1.0 individuals. Seventy-seven participants (82%) completed the study and all assessments (Table 2). Although not mandatory, 66% and 46% completed the weekly food and physical activity logs, for review and feedback from their HC, respectively.

**Table 2.** Demographic characteristics of the participants by program attendance

Demographic Characteristics	Total Sample (*n* = 94) Mean (SD)	Completed at least 50% (≤ 16) Educational Sessions (*n* = 57) Mean (SD)	Completed at least 75% (17-22) Educational Sessions (*n* = 37) Mean (SD)	*P*-Value*
Age (years)	58.8 (12.3)	56.5 (13.5)	62.4 (9.1)	0.02
Baseline A1C	6.8 (1.3)	6.6% (1.1)	7.1% (1.5)	0.09
Baseline weight (pounds)	219.0 (51.4)	212.5 (43.0)	228.9 (61.4)	0.13
Number of people in the household	2.1 (1.0)	1.4 (1.0)	2.0 (1.1)	0.11
Attendance of program sessions	13.7 (6.1)	10.0 (4.9)	19.4 (1.6)	< 0.001

	Number (%)	Number (%)	Number (%)	
Family History of Diabetes	58 (80.6%)	31 (81.6%)	27 (79.4%)	0.52
Family History of Heart Disease	49 (68.1%)	25 (81.6%)	24 (70.6%)	0.48
Diagnosis of Hypertension	61 (71.8%)	39 (76.5%)	22 (64.7%)	0.36
Diagnosis of T2DM	56 (60%)	32 (56.1%)	24 (64.9%)	0.40
Gender (Female)	69 (73.4%)	41 (71.9%)	28 (75.7%)	0.44
Race/Ethnicity				0.78
Non-Hispanic White	67 (93.1%)	36 (94.7%)	31 (91.9%)	
Minorities	5 (6.9%)	2 (5.3%)	3 (8.8%)	
Education				0.21
High School graduate or Technical School degree	27 (30%)	12 (22.6%)	15 (40.5%)	
College or higher	63 (70%)	41 (77.4%)	22 (59.5%)	
Employment Status				0.47
Working full/part time	54 (69.3%)	37 (68.5%)	22 (59.5%)	
On disability	3 (3.3%)	2 (3.7%)	1 (2.7%)	
Retired	25 (27.5%)	11 (20.4%)	14 (37.8%)	
Unemployed	3 (3.3%)	3 (5.3%)	0 (0%)	
Income				0.20
Less than $25,000/year	9 (12.5%)	3 (7.9%)	6 (17.6%)	
$25,000 to $49,999/year	35 (48.6%)	16 (42.1%)	19 (55.9%)	
$50,000 to $74,999/year	14 (19.4%)	11 (28.9%)	3 (8.8%)	
$75,000 or more/year	12 (19.4%)	8 (21.1%)	6 (11.8%)	

* Chi-square for categorical variables, Mann-Whitney U for ordinal, ANOVA for continuous.

### Feasibility metrics

Table 1 presents results of the feasibility metrics. Recruitment, attendance, and retention were excellent with 94 eligible participants recruited over a 6-month period in both study sites. Participants successfully connected with their HC in the first two weeks of program. However, only 72% engaged in weekly discussion of at least 5 or more minutes. Of the 94 participants, four participants dropped out in the first four weeks due to instrumental reasons (e.g., changes in health and mobility, competing family responsibilities, transportation issues, and change in job). The program reached the retention goal (80%) in terms of follow-up A1C test for the 6- (83%) and 12-month (82%) post-intervention assessment. A subset of individuals (*n* = 22) participated in the 3 focus groups (in-person at the church; 77% females, 62.3 years, 59% T2DM) after the program completion for program feedback.

### Program completers and retention

Eighty-two percent of participants completed the program (*n* = 77). Average participation was 13.7 ± 6.1 sessions out of 22 (Table 2). Sixty-three participants (60%) completed at least 50% of the 22 educational sessions and 37 (39%] completed 17 or more (75%) educational sessions. Program attendance was significantly higher among older participants with at least 75% or more educational sessions compared to those who attended at least 50% of the sessions (*p* = .02) (Table 2). Program attendance was higher among those with HbA1C > = 8% (*r* = 0.27; *p* = 0.01), lower BMI (*r* = −0.31; *p* = 0.01), and age (*r* = 0.31; *p* = 0.02), but did not differ by gender or weight loss at 12-months (*p* > 0.05) (Table 3). Estimated distance traveled to attend program was 6.9 ± 5.7 miles by participants (not shown in tables). Participants traveled from several surrounding counties for the program.

**Table 3.** Association of program attention, completion, and outcomes by participant socio-demographic characteristics

	Variables	1	2	3	4	5	6	7	8	9
1	HbA1c^a^	1.0								
2	Mean reduction in weight (lbs)^b^	0.11	1							
3	Gender^c^	0.02	−0.001	1						
4	Age	−0.07	0.10	−0.01	1					
5	BMI	−0.02	−0.11	0.02	−.40**	1				
6	Diabetes Status^d^	.65**	−0.13	−0.005	−0.05	−0.10	1			
7	HbA1c <8% at 12 months	.72**	0.11	0.12	0.04	−0.08	.29**	1		
8	Attendance^e^	0.15	0.03	0.02	.31**	−.31*	0.06	.27*	1	
9	Weight at 12 months	0.11	−0.02	0.16	0.02	−.34*	.28*	−0.07	.22*	1

*. Correlation is significant at the 0.05 level (2-tailed).**. Correlation is significant at the 0.01 level (2-tailed). ^a^ *HbA1c was assessed post-program (12-month assessment).*^*b*^ *Mean reduction in weight (in lbs) was assessed by difference in 12-month weight and baseline value.*^*c*^ *Gender was a categorical variable where 0 = male and 1 = female (reference category).*^*d*^ *Diabetes status was a categorical variable where 0 = individuals with prediabetes and 1 = diabetes (reference category).*^*e*^ *Number of educational sessions attended.*

### A1C

We used an intention-to-treat analysis for assessing A1C. Mean change in A1C over 6- and 12 months as well as chi-square was used to compare the proportion of T2DM participants with an A1C < 8% post-intervention. Eighty-one percent of participants had an A1C < 8% at baseline (none with pre-diabetes, 39.5% of T2DM participants had an A1C > = 8%). In the analyses, with T2DM participants who had at least one follow-up A1C test and at least one encounter with the HC, there were 23.1% of participants with an A1C > = 8% at follow-up (*p* < .001; not shown in tables). A significant association was noted between program attendance and a follow-up A1C test < < 8% at 12 months for participants (*r* = 0.27; *p* = .01; Table 3). In addition, mean reduction in A1C at 12 months was clinically significant for T2DM participants with a reduction of 0.4% compared to their baseline. Similarly, mean reduction in A1C at 12 months was significant for participants who attended 75% or more of the educational sessions (*p* = .04) (Table 4).

**Table 4.** Program changes in glycemic level, weight, and program acceptability by gender, diabetes status, and attendance

	Total	Gender		*P*-value	Diabetes Status		*P*-value	Attendance (Educational Sessions)		*P*-value
	Mean ± SD	Men Mean ± SD	Women Mean ± SD		T2DM Mean ± SD	Prediabetes Mean ± SD		≤ 50% (16) Mean ± SD	≥75% (17-22) Mean ± SD	
*n*	94	25	69		56	38		57	37	
Number of sessions attended	13.7 ± 6.1	13.5 ± 6.1	13.8 ± 6.1	0.82	14.0 ± 5.9	13.3 ± 6.3	0.57	10.0 ± 4.9	19.4 ± 1.5	< 0.001
12-Month Weight change, lbs*	−4.4 ± 11.5	−4.4 ± 6.1	−4.5 ± 9.0	0.95	−5.6 ± 11.0	−2.5 ± 12.4	0.26	−4.71 ± 12.2	−4.04 ± 10.7	0.80
12-Month HbA1c % change**	−0.21 ± 0.47	−0.17 ± 0.32	−0.23 ± 0.52	0.62	−0.41 ± 0.5	−0.07 ± 0.2	< 0.001	−0.13 ± 0.4	−0.35 ± 0.6	0.04
Program benefit	5.4 ± 0.48	5.3 ± 0.54	5.4 ± 0.45	0.54	5.4 ± 0.53	5.5 ± 0.37	0.19	5.3 ± 0.57	5.4 ± 0.33	0.17
Program satisfaction	5.1 ± 0.37	5.2 ± 0.38	5.1 ± 0.37	0.47	5.1 ± 0.40	5.3 ± 0.28	0.03	5.0 ± 0.39	5.2 ± 0.33	0.05
Program barriers	4.3 ± 1.1	4.7 ± 0.67	4.2 ± 1.2	0.08	4.3 ± 1.1	4.4 ± 1.2	0.61	4.0 ± 1.1	4.7 ± 0.95	0.008
Baseline Body Mass Index	36.8 ± 7.7	36.6 ± 6.8	36.9 ± 8.1	0.88	36.9 ± 7.7	37.87 ± 8.7	0.42	39.9 ± 7.73	32.5 ± 5.6	< 0.001

*P-value= difference between groups;* * Weight loss (lbs) was assessed by difference in 12-month and baseline value. A negative estimate indicates greater weight loss. ** HbA1c % change was assessed by difference in 12-month and baseline value. A negative estimate indicates greater reduction in HbA1c value. Program benefit was assessed with a Likert scale (7 items), barriers to session attendance (5 items), and overall program satisfaction (1 item), post-program (range was 1–5). Items were summed with higher score indicating higher benefits and overall satisfaction, and lower barriers. Sample size includes 94 adults with diabetes and prediabetes. Attendance included number of DPM sessions attended in 12 months (range 1–22 educational sessions.

### Weight

We assessed mean change in weight (lbs) from baseline to 12 months. Average weight at the start of the program for participants was 219.1 (51.4) lbs (Table 2). Eighty-one percent of participants had at least one follow-up weight at 12 months. Mean reduction in weight was not clinically significant (defined as less than 5% of body weight) by gender, diabetes status, or program attendance. However, participants with T2DM had a weight loss of 5.6 lbs for, prediabetes 2.5 lbs, 4.4 and 4.5 lbs by males and females, respectively, and 4.7 and 4.0 lbs, by attendance of 50% or 75% educational sessions, respectively (Table 4).

### Program acceptability

A total of 75 participants completed the program benefit, satisfaction, and barrier survey at the end of the program [39] and 22 participants completed the focus groups. The mean benefit, satisfaction, and barriers scores (range 1 = low, 5 high) were 5.4 (0.48), 5.1 (0.37), and 4.3 (1.1), respectively, indicating high benefits and satisfaction and low barriers to program participation. Program benefits were similar by gender, diabetes status, or program attendance. However, higher satisfaction by reported by participants with prediabetes (*p* = .03) and those with higher attendance (*p* = .05; Table 4). Participants who reported higher barriers were significantly less likely to attend educational sessions (*p* = .008; Table 4). Two-thirds (65%) also reported they were successful in making positive lifestyle changes because of the program. In terms of helpfulness of the intervention, educational self-help materials, skill-building exercises, and cooking demonstrations were considered helpful and informative by 70% of the participants. In addition, 72% found the interpretation of blood test results provided to them as informative. It also improved patient empowerment for engagement with family/friends and their healthcare providers for tailored diabetes treatment and self-care. Furthermore, 74% improved their understanding of the risks associated with dysglycemia and how to prevent/manage diabetes. The program improved awareness of dietary tracking, food intake, and healthy diet (79%), improved knowledge about disease and how to control weight (68%), increased physical activity (53%), improved blood sugar monitoring and medication adherence (84%), and a sense of empowerment in terms of managing their chronic conditions (69%).

Program acceptability was also assessed from the three focus groups. These participants reflected the demographic composition – primarily female (77%), NHWs (91%), employed (64%), married (59%), had an income less than $50,000, and T2DM adults (59%). Participants liked the social aspects, such as camaraderie, peer, and HC support, that impacted their program engagement and satisfaction. In addition, the program improved their knowledge, health behaviors, and acceptance of the disease and health risks. One participant stated, “*It’s actually enjoyable thing to actually come and see people and hear their stories and what they’re doing. That kind of helps you, yourself, because you’ll say, golly they’re doing it, you know so I can do it*.” Several participants mentioned setting goals and the act of making a commitment had a positive impact on their continued participation and engagement. Specifically, completing food and activity logs and writing things down seemed to make them feel more accountable for their actions. For example, one participant mentioned, *“That it pays to be honest in regard (sic) to recording your foods and fats and calories, although I do not like it a bit. It helps, it’s a good thing.”* Participants mentioned that diabetes management and treatments vary by person but being able to experience significant positive changes in health outcomes (e.g., reduction in medications, lower A1C) as a result of the program was a motivator. Another participant mentioned, *“My A1C before had been like, you know, 8.5. I went back six months later to my doctor and he took the A1C and it was 7.3. I think he about fell off the chair.”*

There were several program elements that was deemed helpful - receiving the session handouts, feedback from HC on food logs, and weekly discussions that improved their knowledge, healthier eating, and engagement for lifestyle modifications. Support from the HCs for setting goals and having someone to discuss their issues was also described as very helpful (67%). As one participant shared: *“It’s nice to know that there are young people that do care. They take their time out, and when you do talk to them they’re very informative, you know, they do not shove you away, they do not give you short answers, they’re willing to share what they know and help you in any way they can.”* Lastly, the participants felt that they could trust the program leaders and reported feedback on the bloodwork assessments was very helpful. Participants with prediabetes increased their awareness of diabetes complications associated with poor glycemic control which increased their resolve and self-motivation for keeping their blood sugar under control. In terms of program improvements, participants suggested offering more sessions, providing recipes with food demonstrations, and covering more content on how to address dietary challenges. Transportation was not identified as a barrier to program attendance as reported in the literature [40] even during winter months. In addition, participants were not deterred from program engagement due to work priorities, family activities, and personal health issues.

## Discussion

This community-based, multicomponent lifestyle intervention assessed implementation feasibility, acceptability, and program impact on changes in A1C and weight over twelve months among rural adults with T2DM and prediabetes. Our findings suggest that an adapted and culturally tailored DPM program was considered both feasible and acceptable in rural Appalachian settings. By utilizing existing local resources, HCs were able to deliver educational sessions and provide weekly health coaching to develop trust with the participants that was considered particularly vital to keep them engaged throughout the intervention period and improve retention at follow-up. Although health coaching [21,41] and peer support strategies [42] are used to help people maintain healthy behaviors in diabetes and weight loss programs, this is the first community trial to examine the impact of an HC-led DPM intervention in rural Appalachian setting. Qualitative focus groups showed the program was deemed acceptable and benefited both T2DM and pre-diabetes adults. Participants reported health coaching and weekly feedback to be helpful in problem-solving issues and identifying options to stay motivated even during holiday seasons.

Our findings improve our understanding of barriers to program engagement and lifestyle modifications among obese rural adults with T2DM and prediabetes for improved program outcomes (weight loss and A1C). Participants were positive about the program, as demonstrated by their attendance in educational sessions and post-program evaluations. Dropouts after the 1^st^ month were infrequent. Results also provided evidence of successful partnership with faith-based organizations in rural communities. We were also able to demonstrate that churches can support participant recruitment, tailored feedback on curriculum, and are uniquely equipped to provide public space for educational sessions, kitchen for cooking demonstrations, easy parking, and availability during weekday evenings & weekend afternoons for community-based interventions in rural communities.

As might be expected, initial positive improvements in health behavior (e.g., dietary intake and physical activity) at 6 months declined by end of the program. However, changes in A1C were notably clinically significant for T2DM adults at 12 months. Although the average weight loss showed none of the participants achieved the 5% weight loss goal that is promoted by NDPP and other lifestyle programs, however, participants were proud of losing even a few pounds and/or when they were able to maintain their initial weight loss success. In addition, weekly health coaching provided participants to build relationships, set short-term goals, monitor/track their diet/physical activity which had a positive effect on health behavior change, and advocate for tailored self-care with providers. In a healthcare setting, it is the patient’s responsibility to proactively connect/follow-up with patient educators (e.g., nurses, health educators, and patient navigators) to promote their health and well-being, and improve self-care. However, barriers to these interactions for rural patients are numerous and include insufficient referrals, socioeconomic factors (e.g., poverty, transportation, low educational level), poor access, inadequate knowledge of disease, and high cost [43–47].

In terms of retention, 82%participants completed their 12-month program sessions and post-program assessment. This retention rate is higher than other community-based lifestyle interventions in WV including the NDPP lifestyle program and mobile Health interventions [48,49] as attrition rates are generally high (∼50%). Cannon and his colleagues analyzed 41,203 individuals enrolled in the CDC-recognized Diabetes Prevention Recognition Program (in-person) from January 2012 to February 2017. The median retention reported for the NDPP was 28 weeks and pooled retention showed 63.1% of participants were retained through the first 6 months (18^th^ week) and 31.9% at 44 weeks. It should be noted that the study duration of the DPM is similar to the NDPP with weekly sessions for the first 12 weeks, biweekly sessions for next 2 months, and monthly sessions for the last 6 months. Similar to the NDPP, our retention rates were higher for older participants and those with higher A1C but did not differ by gender or weight loss [48]. We envisage weekly health coaching and successful interaction of participants with HCs and program peers to improve program engagement and retention. In addition, the group-based educational sessions allowed co-learning from peers who became members of their social network. Program attendance was positively associated with the program acceptance and satisfaction as reported in survey and focus group findings. Higher attendance was associated with prevention or delay of diabetes and its complications in NDPP randomized controlled trial [50].

Diabetes is often associated with micro and macrovascular complications in rural WV and Appalachian adults [51]. While the Affordable Care Act improved health insurance coverage for approximately 10% of T2DM adults in WV, diabetes clinical care and self-management did not improve between 2010 and 2014 [52]. Hence, follow-up A1C assessments provided to the participants can be promising and DSME/S interventions could incorporate free A1C assessments for monitoring and glycemic control [38]. The poor health outcomes of rural adults with chronic conditions are often because few programs are available and patients who live in geographically isolated communities such as in WV. Individuals are required to travel long distances and have reported barriers such as low literacy, knowledge, and financial worries that have resulted in T2DM being considered a “family” rather than “an individual” disease in Appalachia [26,53]. In addition, the majority of WV’s 55 counties are classified as rural “isolated small” and “small” and medically underserved areas [6]. Hence, food deserts are a symptom of the broader low socioeconomic conditions as T2DM, and prediabetes individuals endure limited food access [54]. Our findings suggest that despite these barriers, creative strategies can improve knowledge and health behavior among T2DM individuals with comorbidities (or multimorbidity) that are highly prevalent in WV (42% vs 30% nationally) making self-care more complex and challenging[55,56]. Hence, utilizing a network of organizations and providers for community-based programs to empower individuals to manage their disease and prevent complications is important for lowering the health and economic burden of the disease.

This study builds on the research team’s success with culturally tailored DPM programs designed for rural adults. Regarding acceptability, participants reported that program components were helpful in improving their knowledge and self-confidence. The social aspects of the program and the non-judgmental environment provided them with opportunities to ask question, share their challenges and success, and learn from others. HCs played a valuable role in providing support and behavior change strategies which may have contributed to maintaining engagement, retention, and program satisfaction. The Appalachian culture of distrust and healthcare-avoidant behaviors should be considered for lifestyle programs including concerns reported by participants (e.g., meal recipes, food demonstrations, and daily regimen challenges) for maintaining patient engagement and retention. The overall acceptance of the HCs was expected given the demonstrated effectiveness of CHW and health coaching in diabetes care [57,58]. However, HCs could be integrated into healthcare teams to serve as a bridge between patients and providers and/or provide culturally tailored patient education to improve patient-provider relationships. Lastly, as with any pragmatic trail that utilizes CBPR approach, there needs to be a balance between study design and respect for the community we want to support.

## Limitations

There were several limitations in this study. First, we were not able to detect statistically significant differences in program outcomes (A1C and weight loss) at 12-month follow-up. However, the findings from this study are promising and warrant a larger, pragmatic clinical trial for a comparative effectiveness of DPM versus individual NDPP or diabetes self-management interventions independently. Second, a time-series evaluation was conducted with no usual care control group for comparison. The 12-month intervention did not allow for a wait-listed or usual care group, commonly used as a comparator to examine intervention effectiveness. However, the study design was supported by the stakeholders who considered the program as pragmatic for rural participants and deemed having a control arm with no educational sessions as unethical. In addition, a wait-listed intervention group that offered all participants the opportunity to participate was not feasible due to the 12-month program duration. Also, health behavior, program benefits, barriers, and satisfaction were self-reported, plausibly introducing recall bias and social desirability bias. Study assessments were completed at the church and during a weekend that contributed to missing A1C for participants who had family emergencies, prior commitment, and lack of transportation for that weekend. Third, given that rural participants have higher social determinants of health issues (e.g., housing insecurity, food insecurity, etc.), it is possible that the program may not have addressed them adequately. Lastly, the generalizability of our findings may be limited due to the small sample size, and we had few minority participants (African American, Asian, and Hispanic) due to predominantly NHWs (97%) in the state.

## Lessons learned

From a translational research and public health education perspective, it is important to keep in mind that individuals with prediabetes and T2DM may be experiencing mistrust, discrimination, and social determinants of health issues in rural areas. Therefore, our study team learned some valuable lessons from both the CAB and the participants – that it was critical to use culturally appropriate communication methods, be flexible in scheduling the educational sessions and assessments, and provide accessible program locations in order to meet participants where they were. In addition, HCs applied an equity lens to address challenges to healthy food by educating them on how to buy food with a low budget, meal planning, use discount grocery stores, shop generic and buy in bulk or frozen food for longer shelf life. This highlights the importance of resourcefulness and empowering participants to improve their health and well-being with resource limitations and food options. Furthermore, we recognized that implementation of this trial relied on strong, reciprocal partnerships among the study team, community stakeholders, and faith-based organizations. The academic-community partnership was particularly strengthened by the HCs who served as a bridge between the patients and their providers to advocate for tailored care management [59]. Lastly, striking a balance between intervention design and community respect is crucial in pragmatic trials that utilize a community-based participatory approach and benefit our support for improved access to educational programs.
